# The Heel-Jerk

**Published:** 1903-08-29

**Authors:** 


### THE HEEL-JERK.

The clinical significance of suppression or exaggeration of the knee-jerk has long been generally appreciated, and the physiological mechanism on which the jerk depends is well understood. A knowledge of that mechanism naturally suggests that any muscle, when put upon the stretch, will contract in response to a sharp blow inflicted over its tendinous insertion, and this, as a matter of fact, is found to be the case. The anatomical position and relationships of certain muscles allow these necessary conditions to be readily established, and thus a biceps-, triceps-, and wrist-jerk can be obtained in the upper limb, and in a similar fashion a jaw-jerk can be produced. In the lower limb, the tendo Achillis offers itself as convenient for this form of mechanical stimulation, and during the last two or three years considerable attention has been paid to the movement so produced—the heel-jerk, as it may be called. This is readily elicited if the patient is made to kneel on a chair, and, whilst some pressure is exerted on the sole in order to make the calf-muscles tense, a sharp blow is delivered over the insertion of the tendon into the os calcis. The response is a quick flexion of the foot exactly comparable to the knee-jerk. Even with a patient confined to bed the jerk may be readily obtained if care is exercised. What is essential is to flex the knee and to make the calf muscles tense by slight pressure on the sole. In a very exhaustive study of the subject Dr. Edwin Bramwell has established a number of valuable conclusions. First, on the basis of a large number of observations, it is shown that the heel-jerks are, with a few very doubtful exceptions, constantly present in persons under 50 years of age. After that age the jerks become less active, and hence their absence in elderly people cannot confidently be declared abnormal. Secondly, as would naturally be anticipated, the circumstances which lead to loss of the knee-jerks, *e g.*, tabes dorsalis, peripheral neuritis, etc., lead also to disappearance of the heel-jerks. But, and this is the point of main practical importance, it is possible for the latter to be lost whilst the knee-jerks retain their normal activity. Indeed, in tabes dorsalis it is the rule for the heel-jerks to be diminished or to disappear before the knee-jerks. Obviously this observation may be of definite value in assisting in the early diagnosis of an otherwise doubtful case. Pains in the limbs, a feeble pupil response to light, recurring attacks of gastric pain and vomiting, some difficulty in micturition—these and certain other events, though recognised as suspicious, are apt to be more or less lightly interpreted so long as the knee-jerks are found to be active. But it is now manifest that absence of the heel-jerks in such circumstances is a fact hardly less significant than loss of the knee-jerks, and its frequently earlier occurrence gives the opportunity for a correspondingly earlier diagnosis. A similar statement may be made in regard to general paralysis of the insane and to peripheral neuritis. The diagnostic value of the heel jerk extends also in another direction. Babinski has shown that in sciatica it is usually lost on the affected side. Its absence therefore proves the existence of a definite sciatic neuritis and so assists in the distinction of this condition from the hysterical form of the disease. Once lost, as for example from sciatica, the heel-jerk may remain absent for years after the sciatica has disappeared.

